# MicroRNA-10a promotes granulosa cells tumor development via PTEN-AKT/Wnt regulatory axis

**DOI:** 10.1038/s41419-018-1117-5

**Published:** 2018-10-22

**Authors:** Jiajie Tu, Hoi-Hung Cheung, Gang Lu, Zijiang Chen, Wai-Yee Chan

**Affiliations:** 1 School of Biomedical Sciences, The Chinese University of Hong Kong, Faculty of Medicine, the Chinese University of Hong Kong, Hong Kong SAR, China; 2 CUHK-SDU Joint Laboratory on Reproductive Genetics, School of Biomedical Sciences, the Chinese University of Hong Kong, Hong Kong, China; 3 Institute of Clinical Pharmacology, Anhui Medical University, 230000 Hefei, Anhui China; 4 National Research Center for Assisted Reproductive Technology and Reproductive Genetics, 250001 Jinan, Shandong China; 5 Center for Reproductive Medicine, Shandong University, 250001 Jinan, Shandong China

## Abstract

We have previously reported that microRNA-10 family could disturb normal development of granulosa cells (GC) during follicle formation. In the current study, the effect of miR-10a on granulosa cell tumor (GCT), a subtype of ovarian cancer, was examined. Strong miR-10a signal was detected in tissues from malignant GCT patients. Forced expression of miR-10a significantly promoted cell proliferation, migration, invasion, ovarian hormone production, and repressed anticancer drug-induced apoptosis in vitro. The oncogenic role of miR-10a was further validated in an orthotopic GCT model in vivo. In addition, by using CRISPR-Cas9 system, the aggressive phenotype was repressed in miR-10a knockout cancer GC. By using a heterotopic mice model, the oncogenic role of miR-10a was confirmed in vivo. RNA-seq, FISH, western blot, luciferase reporter assay were used to identified PTEN, a well-known anti-GCT gene, as direct functional target of miR-10a in cancer GC; Akt and Wnt were also found as two associated oncogenic pathways of miR-10a in cancer GC. Taken together, our results demonstrate that the miR-10a could promote GCT development via synergistically regulating PTEN, Akt, and Wnt pathways.

## Introduction

Ovarian cancer is the sixth most commonly diagnosed cancer and the fourth cause of death in women with malignancy worldwide^1^. There are three major types of human ovarian cancers: epithelial, granulosa cells (GC), and germ cell ovarian cancer. The majority of ovarian cancers is epithelial ovarian cancer (EOC) and most ovarian cancer-related studies focus on it^2^. However, the etiology and mechanism about another form of ovarian cancer, granulosa cell tumor (GCT) are still largely unknown. The molecular pathways that regulate GCT development have not been fully understood, posing a challenge to improving clinical outcome. Hence, elucidating the underlying molecular mechanism driving GCT progression is crucial for the development of targeted therapy that can help improve survival outcomes in patients.

MicroRNAs (miRNAs) are a class of small non-coding RNA molecules that negatively regulate gene expression at post-transcriptional level, primarily via base pairing to the 3′-untranslated region (3′UTR) of the target messenger RNA transcripts^3^. miRNAs have been shown to play a critical role in the initiation and development of various types of cancers through regulating important target genes and modulating signaling pathways^4^. A number of miRNA profiling studies have identified miRNAs associated with chemotherapy resistance and disease progression in EOC^5^. Interestingly, by analyzing two studies of advanced ovarian cancer specimens from The Cancer Genome Atlas (TCGA) (http://cancergenome.nih.gov)^6^, we found consistent amplification of miR-10a which was positively associated with lower overall survival rate during ovarian cancer pathogenesis. Furthermore, we found miR-10a was highly expressed in malignant GCT. From our previous study, we showed miR-10 family could repress normal development of GC during folliculogenesis in ovaries^7^, indicating a potential crosstalk of miR-10 in regulating the pathological condition of GC.

The recent studies analyzing a large number of high-grade GC cancer samples suggest that the acquisition and development of GCT are accompanied by the activation of AKT and Wnt pathways associated with poor patient outcome^8–10^. PTEN, the well-known anti-cancer gene, is also proved as a tumor repressor in GCT^11^. The molecular event driving PTEN and AKT/Wnt pathways in GCT, however, remains poorly understood. In this study, we identified that miR-10a could promote development of GCT both in vitro and in vivo via modulating PTEN-AKT/Wnt axis in GCT. Our data highlight a functional role for miR-10a in GCT biology and uncover a potential prognostic biomarker and molecular target for the treatment of GCT.

## Results

### MiR-10a is overexpressed in ovarian tumors and GCTs

**Fig. 1:**
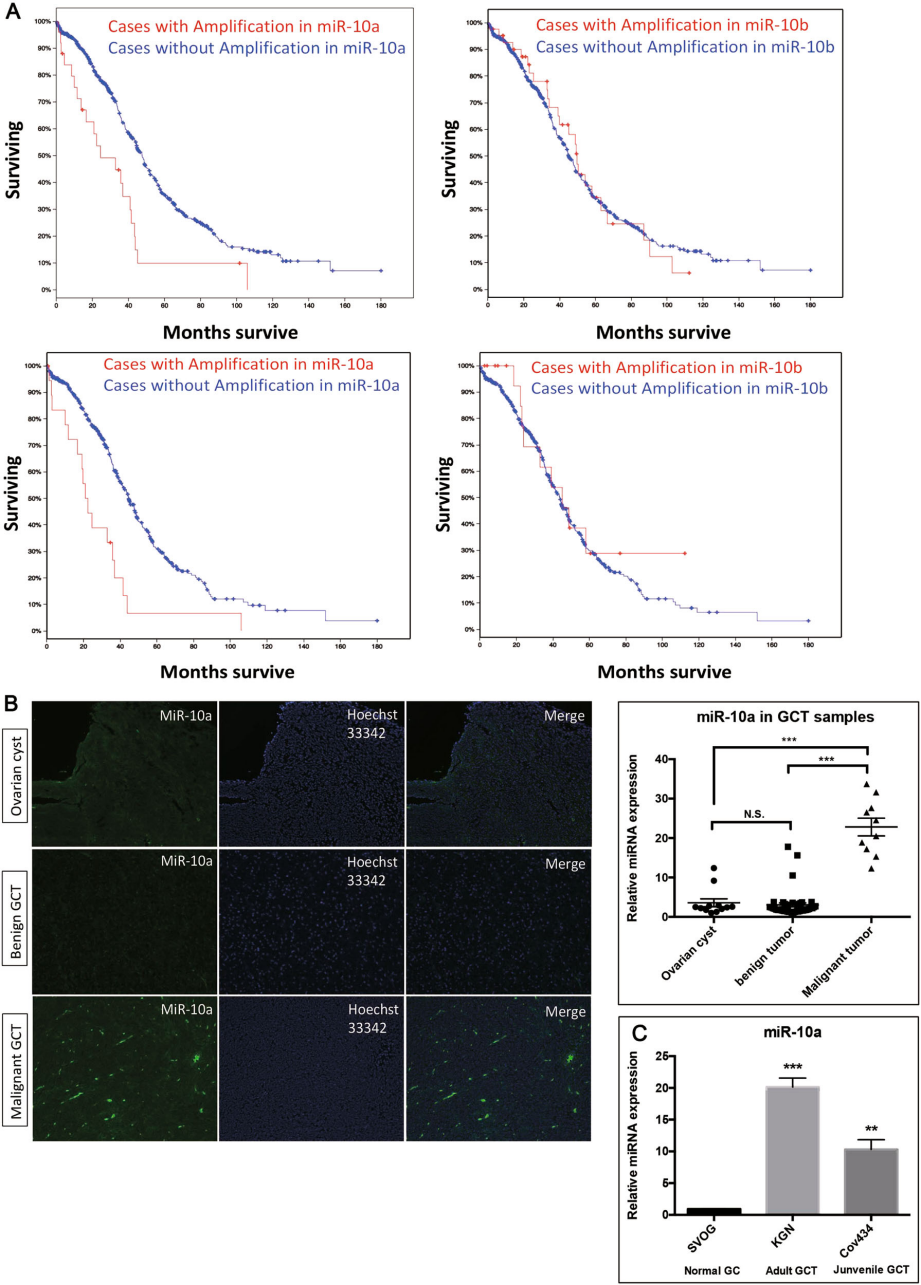
MiR-10 in ovarian cancer patients and granulosa cells tumor and cell lines. **a** Above and below are two independent groups of ovarian cancer patients. Lower survival rate of ovarian cancer patients for those cases with amplification of miR-10a, but not miR-10b. **b** Fluorescence in situ hybridization (FISH) studies detected a stronger staining in malignant GCT tissues than either begin GCT tissues or ovarian cysts. **c** The level of miR-10a increases in two GCT cell lines KGN and Cov434. Each bar in the figure represents the mean ± SEM of triplicates. ** *P* < 0.01 and *** *P* < 0.0001

GCT is a subtype of ovarian tumor. To assess the role of miR-10a in GCT, we compared the overall survival rate of ovarian cancer patients from the two datasets of TGCA. The result showed that the survival rate was much lower in those cases with miR-10a amplification than those without alteration of miR-10a (Fig. 1a). This difference was not seen for miR-10b. We observed similar results from the two independent cohorts of ovarian cancers. To investigate whether miR-10a is associated with GCT malignancy, fluorescence in situ hybridization (FISH) was performed to examine miR-10a expression in banked biopsies from GCT patients. FISH results indicated that malignant GCT expressed more miR-10a than benign GCT or ovarian cyst (*P* < 0.0001) (Fig. 1b). The levels of miR-10a in two GCT cell lines KGN and Cov434 (KGN is an adult GCT line and Cov434 is a juvenile GCT line) were also significantly up-regulated compared to SVOG, a normal human granulosa cell line (Fig. 1c). Taken together, these observations suggest that miR-10a amplification is an indicator of poor prognosis of ovarian cancer. Up-regulation of MiR-10a was found in malignant GCT tissues and GCT cell lines, signifying an oncogenic role for miR-10a in GCT.

### MiR-10a promotes GCT tumorigenesis in vitro and in vivo

**Fig. 2:**
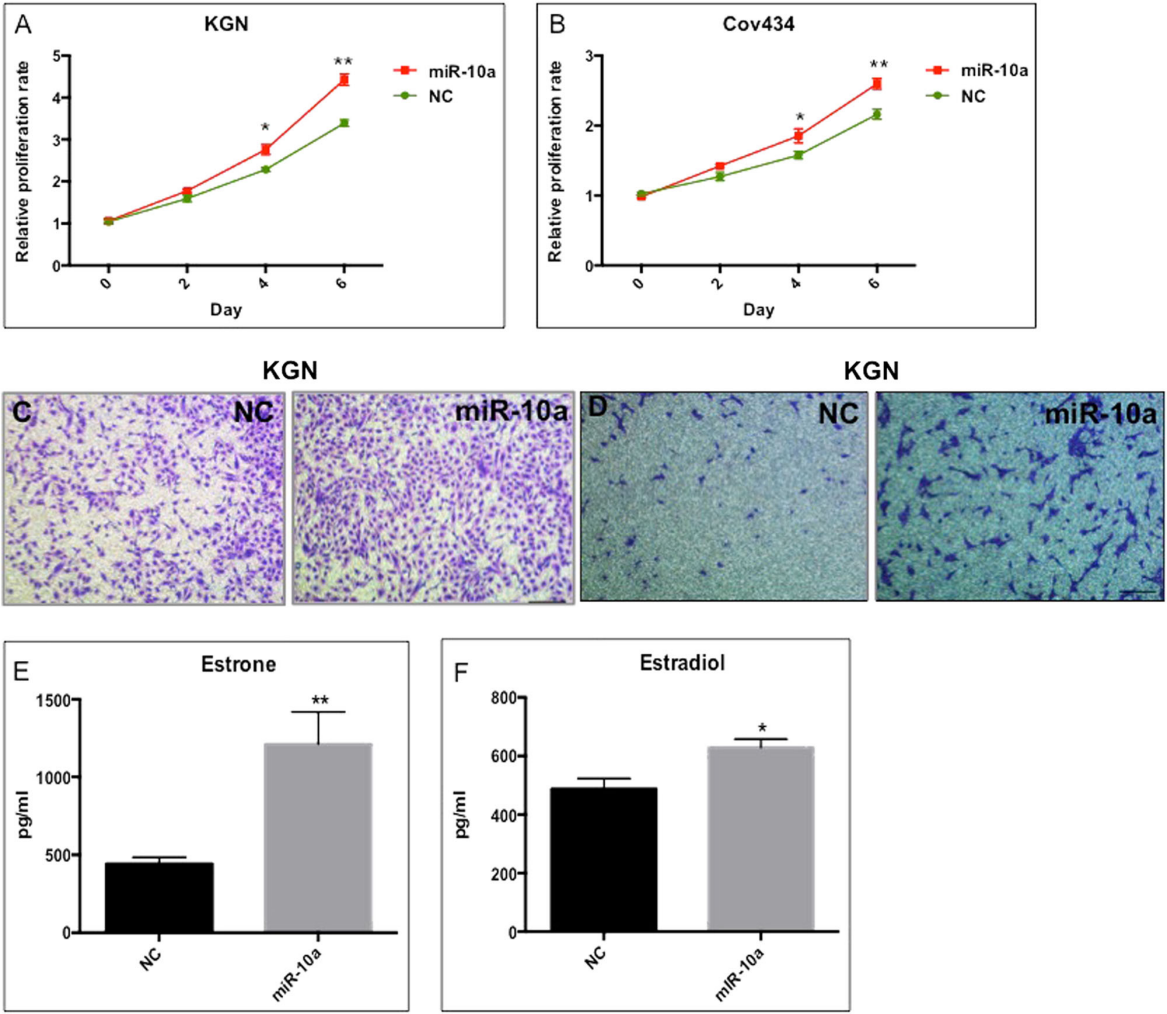
The effect of miR-10a overexpression on cancer GC in vitro. **a**, **b** Proliferation of GCT cells was induced by miR-10a mimics. **c** MiR-10a overexpression induced migration of KGN cells. **d** MiR-10a overexpression induced invasion of KGN cells. **e**, **f** Estrone and estradiol produced by GCT cells were induced by miR-10a. Each bar in the figure represents the mean ± SEM of triplicates. *P < 0.05 and ** *P* < 0.01

**Fig. 3:**
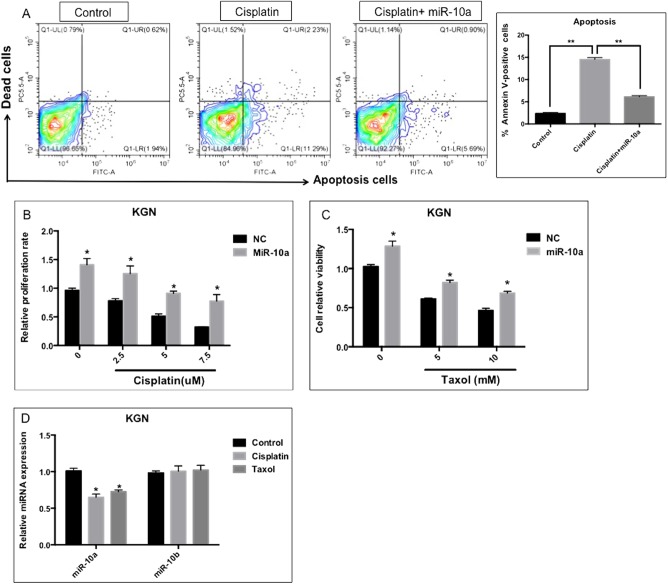
MiR-10a promotes chemoresistance of KGN cells in vitro. **a** MiR-10a could repress the cisplatin-induced apoptosis in GCT cells. **b**, **c** Cisplatin-induced or taxol-induced cell death was ameliorated by miR-10a. **d** Cisplatin treatment decreased endogenous expression of miR-10a, but not miR-10b in KGC cells. Each bar in the figure represents the mean ± SEM of triplicates. *P < 0.05

To investigate the potential role of miR-10a in GCT development, we first determined its effects on cell proliferation, migration, and invasion in vitro. When KGN and Cov434 cells were transfected with miR-10a mimics (Supplementary fig. 1A), there was an increase in cell proliferation (Fig. 2a, b). Transwell migration and invasion assays showed that miR-10a overexpression significantly enhanced GCT cellular motility and invasion (Fig. 2c, d). Estrone and estradiol are female hormones secreted by GCs. High level of these two hormones is found in GCT patients. We thus performed ELISA to evaluate the effect of miR-10a on production of estrone and estradiol. The results showed that miR-10a significantly increased the production of these two hormones by GCT (Fig. 2e, f). Importantly, miR-10a could modulate the chemoresistance of GCT, as indicated by less apoptotic cells induced by anticancer drug cisplatin (Fig. 3a). MiR-10a also enhanced cell survival when treating cells with cisplatin or taxol in a dose-dependent manner (Fig. 3b, c). Moreover, cisplatin and taxol treatment decreased miR-10a, but not miR-10b level (Fig. 3d). This observation was consistent with the finding that miR-10b lacks association with the survival rate of ovarian cancer patients (Fig. 1a). In summary, miR-10a overexpression promotes proliferation, migration/invasion, and chemoresistance of GCT cells in vitro.

**Fig. 4:**
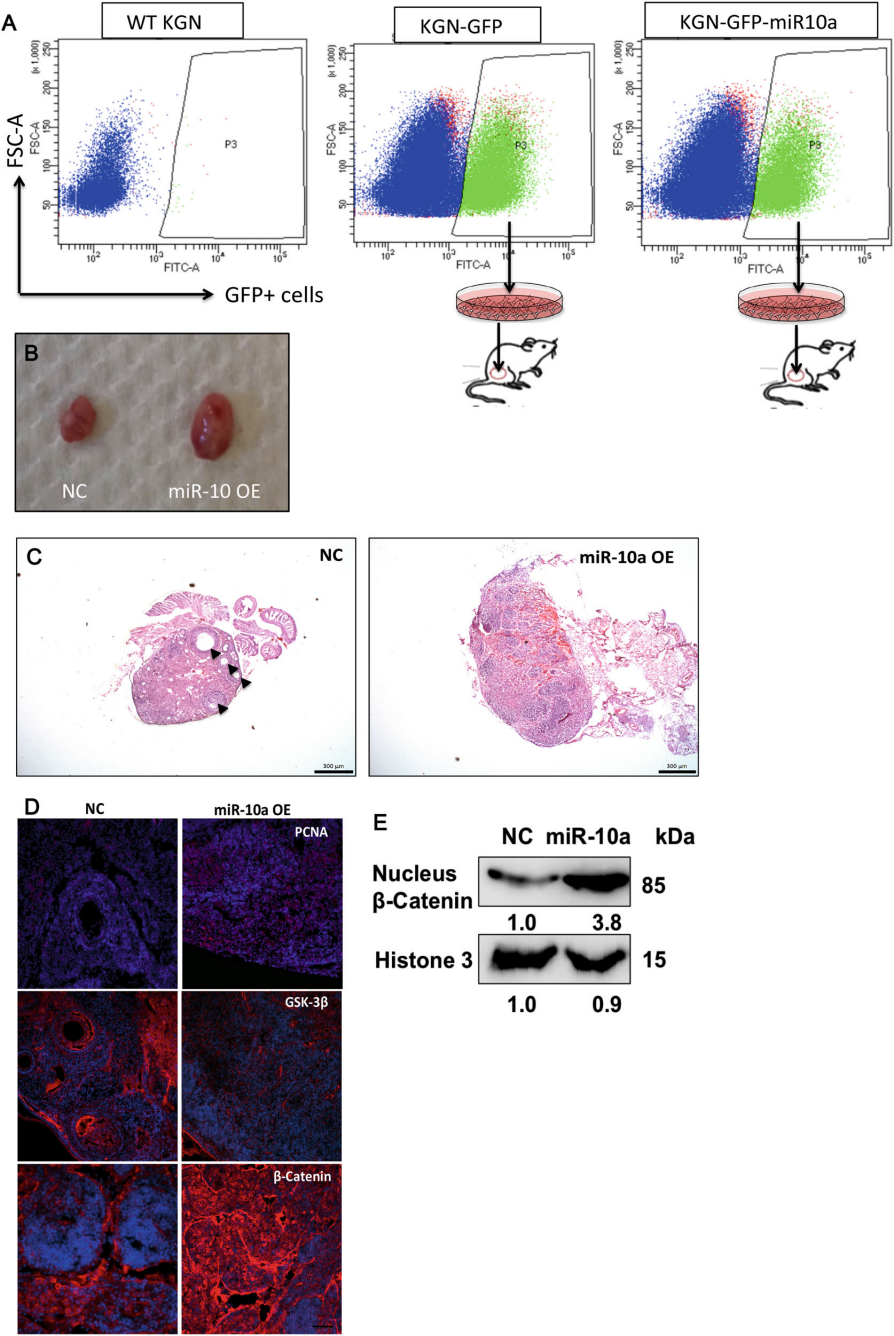
The effect of miR-10a overexpression on cancer GC by using an orthotopic model. **a** Stable miR-10a OE in KGN cells was established by GFP sorting. **b** The tumor burden of KGN cells OE miR-10a was larger than that of control KGN cells (*n* = 5). **c** H&E staining for histological analysis of miR-10a OE KGN cells and NC KGN cells injected ovaries (triangles indicated some normal follicles still could be found in NC KGN cells injected ovaries). **d** The effects of miR-10a on proliferation marker PCNA and two Wnt components (GSK-3β and β-Catenin) in control KGN cells and miR-10a OE KGN cells-formed tumor. **e** The effect of miR-10a on nuclear β-Catenin in control KGN cells and miR-10a OE KGN cells-formed tumor. OE overexpression

We next used an orthotopic model to evaluate the role of miR-10a on GCT in vivo. Stable miR-10a overexpression in KGN cells (Supplementary fig. 1B) was established by retrovirus transduction followed by GFP^+^ sorting (Fig. 4a). 1 × 10^6^ KGN cells were directly injected under the ovarian bursa. After 4 weeks, miR-10a overexpressing KGN cells-formed tumor showed more rapid growth rate than that of control KGN cells (Fig. 4b). Histological analysis demonstrated that several normal follicles still could be found in control KGN cells-injected ovary, while there was no normal follicle presence in miR-10a OE KGN cells injected ovary (Fig. 4c). PCNA, a marker of cell proliferation, was elevated in miR-10a OE tumors (Fig. 4d). It is reported that Wnt pathway was activated in GCT, therefore we also evaluated the expression of GSK-3β (blocker of Wnt pathway) and β-Catenin in control and miR-10a OE KGN-formed tumors. Immunostaining results showed that GSK-3β was repressed and β-Catenin was activated in miR-10a OE KGN-formed tumor (Fig. 4d). In addition, nuclear β-Catenin was also induced by miR-10a (Fig. 4e), validating that Wnt pathway was activated by miR-10a in GCT. All together, our results suggest that miR-10a could promote GCT tumorigenesis and induce Wnt pathway activation in vivo.

### The related pathway and target of miR-10a in GCT

**Fig. 5:**
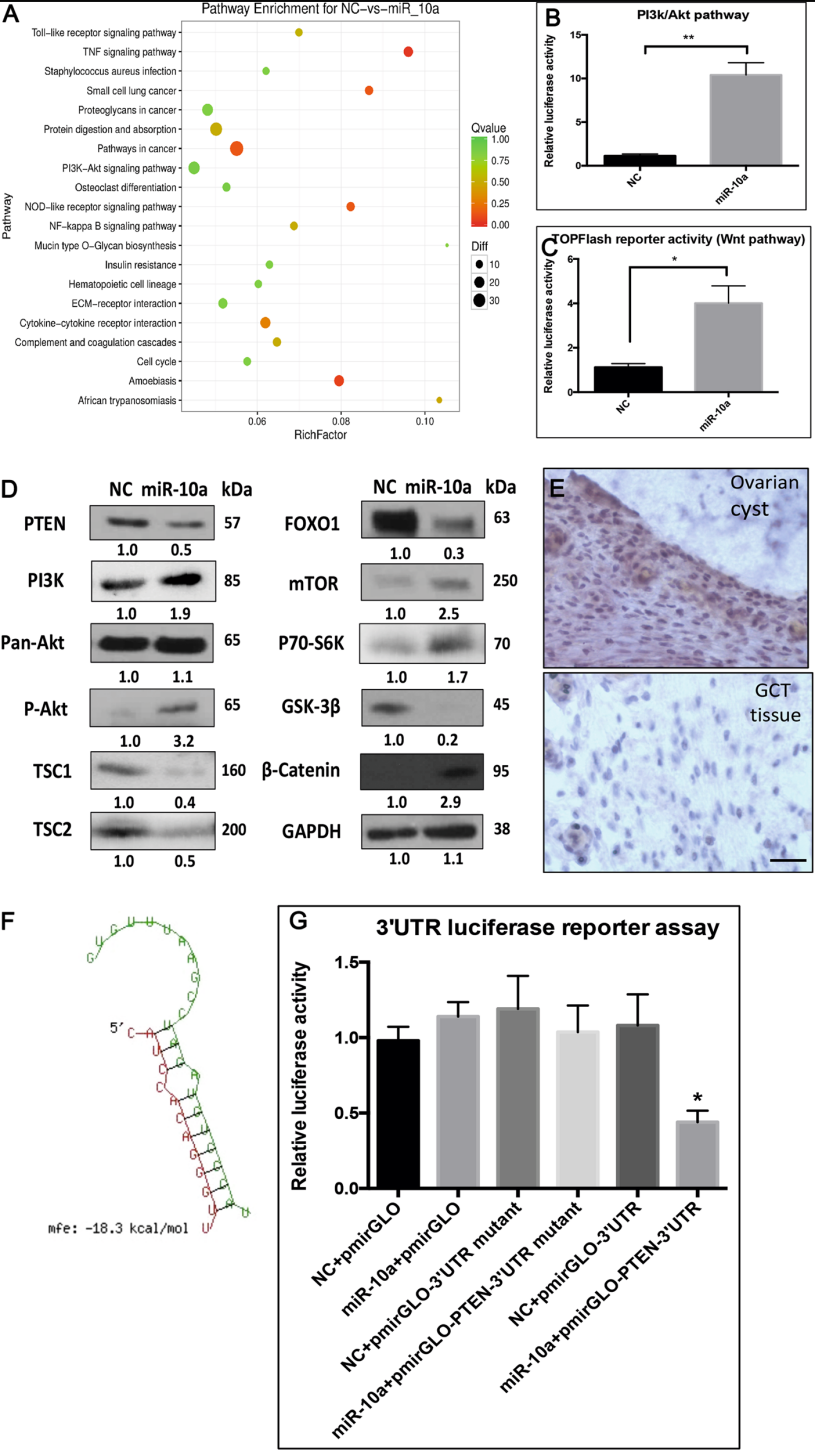
The effect of miR-10a on KGN cells at transcriptome level. **a** Signaling pathway enrichment analysis of miR-10a-OE in GCT. **b**, **c** Pathway reporter assay showed that miR-10a could regulate PTEN, PI3K-AKT, and Wnt signaling in GCT. **d** MiR-10a could modulate several key factors within AKT and Wnt pathways. **e** PTEN expression in GCT tissues by IHC. **f** The predicted binding site between PTEN 3′UTR and miR-10a seed sequence. **g** Luciferase reporter assay showed that miR-10a could directly binding to 3′UTR of PTEN. Each bar in the figure represents the mean ± SEM of triplicates. *P < 0.05 and ** *P* < 0.01

To identify the direct targets of miR-10a and the associated pathway in GCT, we analyzed the transcriptomes of miR-10a OE KGN and control KGN cells by RNA-seq (Supplementary fig. 2A). Signaling pathway enrichment analysis revealed the association of miR-10a with several cancer- related pathways (Fig. 5a). Pathways such as PI3K-Akt, NF-kB, and Wnt signaling have been known to be critical in GCT^12, 13^. To confirm the relevance of these pathways to miR-10a, we performed pathway reportor assay and found that miR-10a could activate PI3K-Akt and Wnt signaling in GCT (Fig. 5b, c). In addition, Western blot results further confirmed the change of several key factors, including PTEN, PI3K, AKT, TSC1, TSC2, mTOR, FOXO1, S6K, GSK-3β, β-Catenin, within these two pathways (Fig. 5d).

We next sought to identify the binding target(s) of miR-10a for modulating its function. Integrated analysis of the RNA-seq data with miRNA target prediction software, PTEN was predicted to be a potential direct target of miR-10a in GCT. A putative miR-10a binding site at the 3′-UTR of PTEN is shown (Fig. 5f). Immunohistochemistry (IHC) analysis showed decreased level of PTEN protein in ovarian GCT tissues (Fig. 5e). The inverse expression patterns of PTEN and miR-10a in GCT tissues suggest a negative regulatory effect of miR-10a on PTEN. Indeed, PTEN was significantly reduced by miR-10a in GCT, as validated by Western blot (Fig. 5d). To determine whether PTEN is a direct target of miR-10a, the 3′ UTR containing the putative miR-10-binding site was cloned into a luciferase reporter. Consistently, luciferase activity was repressed by transfecting miR-10a mimics (Fig. 5g). These results indicated that PTEN was a direct target of miR-10a in cancerous GC.

### Knockout (KO) miR-10a in cancerous GC by CRISPR-Cas9

**Fig. 6:**
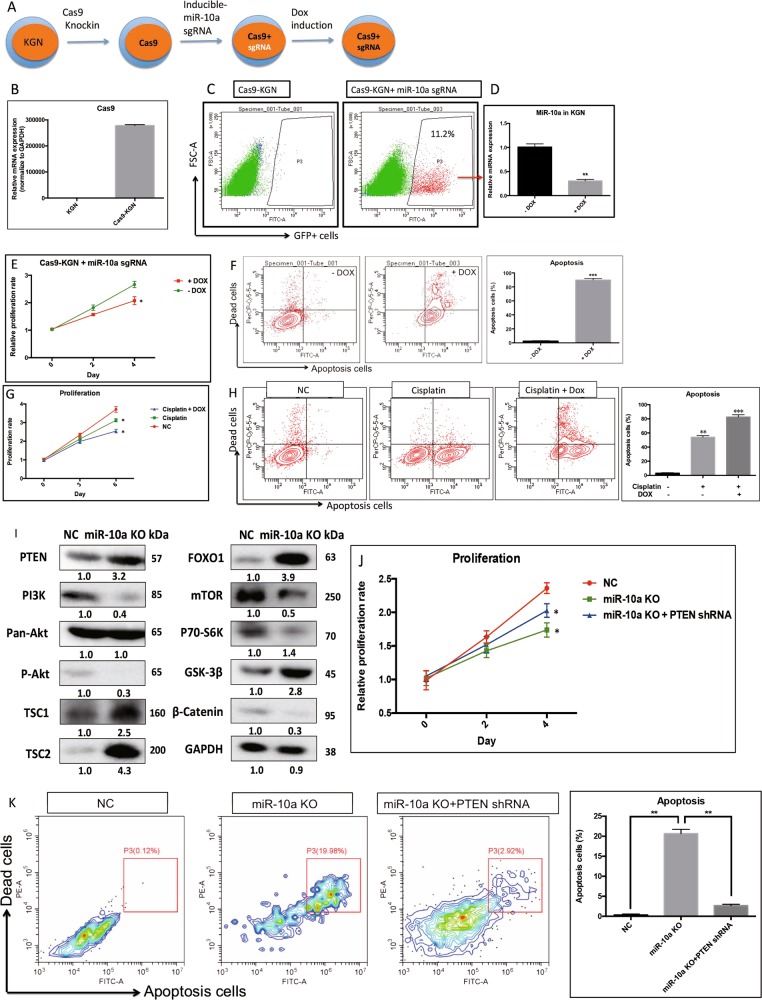
Knockout miR-10a in cancer GC by CRISPR/Cas9. **a** Schematic diagram showing the strategy to construct dox-inducible miR-10a knockout KGN cells. **b**–**d** Validation of the inducible CRISPR/Cas9 system in KGN cells. **e** Proliferation was repressed in miR-10a KO cancerous GC. **f** Apoptosis was induced in miR-10a KO cancerous GC. **g**, **h** MiR-10a KO promoted cisplatin-induced cell proliferation arrest and cell death in cancer GC. **i** The expression of PTEN-Akt/WNT pathway in miR-10a KO cancerous GC. **j**, **k** PTEN knockdown rescue proliferation and apoptosis in miR-10 KO cancerous GC. Each bar in the figure represents mean ± SEM of triplicates. *P < 0.05 and ** *P* < 0.01

We have demonstrated gain-of-function for miR-10a using miRNA mimics. To demonstrate loss-of-function of miR-10a in cancerous GC, we applied an inducible CRIPSR-Cas9 system to generate miR-10a KO^14^. We first generated stable Cas9-expressing KGN cells by transducing Cas9-mCherry lentivirus (Fig. 6a, b). Then we validated the mutagenesis activity of the Cas9/CRISPR by transducing cells with GFP-tagged, dox-inducible sgRNA lentivirus targeting the genomic locus of miR-10a precursor. Dox treatment did not affect WT KGN cells proliferation and apoptosis, which exclude the possible leakage effects of Dox (Supplementary fig. 2B and 2C). After GFP sorting (Fig. 6c), induction of sgRNA expression by dox led to efficient loss of miR-10a expression (Fig. 6d). We next tested the effect of miR-10a KO in cancerous GC in vitro. In line with the gain-of-function studies, miR-10a KO resulted in slower cell proliferation (Fig. 6e). Apoptosis, as expected, was increased in miR-10a KO cancerous GC (Fig. 6f). In addition, miR-10a KO sensitized cells to anti-cancer drug and promoted cisplatin-induced cell proliferation arrest (Fig. 6g) and apoptosis (Fig. 6h). Western blot was performed to detect the expression of PTEN/Akt/WNT pathway in miR-10a KO cancerous GC (Fig. 6i). To further prove that the oncogenic effect of miR-10a on GCT is through PTEN, a PTEN shRNA lentivirus was used to knockdown PTEN in miR-10a KO cancerous GC (Supplementary Fig. 2D). After PTEN knockdown, the repressed proliferation was restored in miR-10a KO cancerous GC (Fig. 6j), which was validated by apoptosis detection (Fig. 6k). These results together demonstrated an oncogenic role of miR-10a/PTEN regulatory asix in GCT tumorigenesis in vitro.

### The miR-10a KO inhibits tumor formation in vivo

**Fig. 7:**
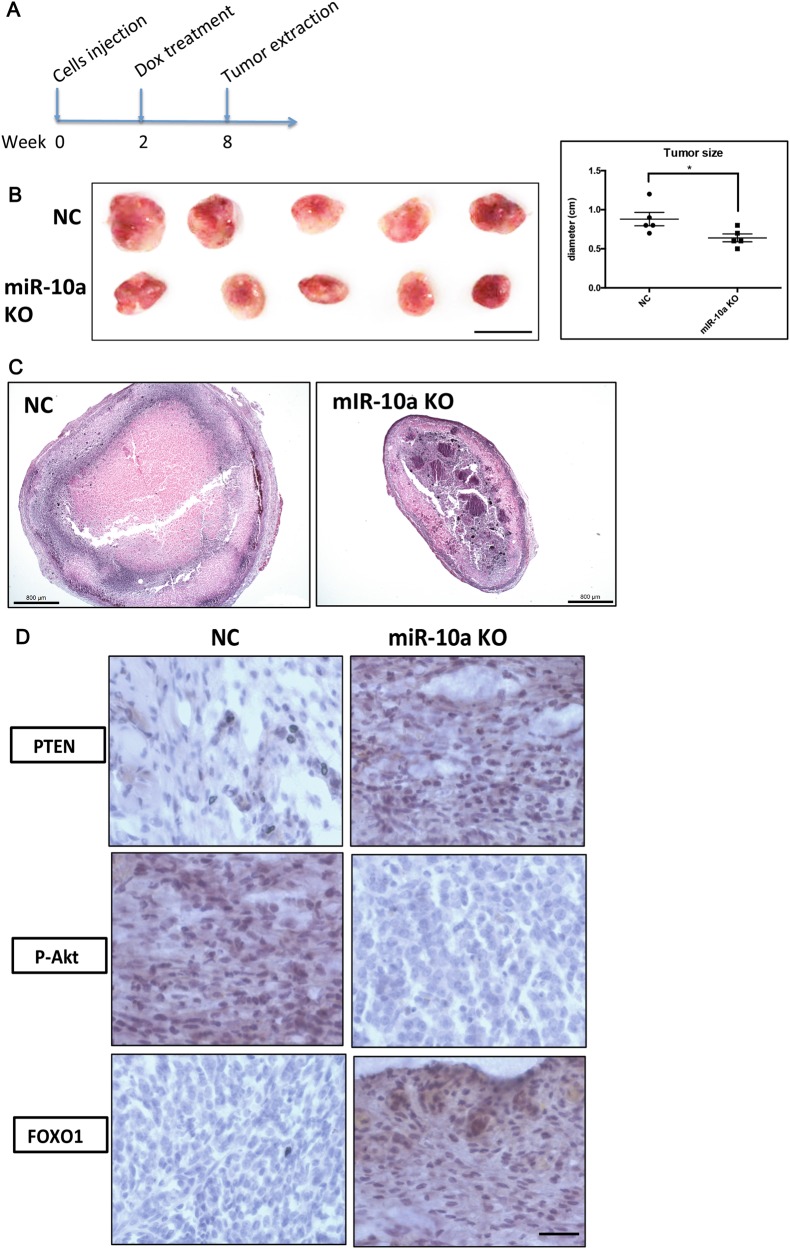
The effect of miR-10a KO on KGN cells-formed tumor in vivo. **a** Timeline showing the process of establishing the GC heterotopic model. MiR-10a KO KGN or control KGN cells were injected in the back of nude mice, followed by feeding mice with dox to induce CRISPR/Cas9-mediated miR-10a KO. **b** The size of tumor formed from miR-10a KO KGN cells was smaller in dox-treated group (*n* = 5). **c** Histological analysis of the tumors formed from miR-10a KO cancer GC and control cancer GC. **d** IHC staining showed the expression of tumor suppressor PTEN, P-AKT, and FOXO1 in the miR-10a KO and control tumors

We subsequently assessed the effect of miR-10a KO on GCT formation in vivo. We utilized a cancerous GC ecptoic model wherein the transgenic KGN cells were subcutaneously injected into nude mice, followed by dox treatment after 2 weeks of tumor transplantation (Fig. 7a). Tumors grown in dox-treated mice had a smaller size, compared with the untreated mice (Fig. 7b). Histological analysis showed increased angiogenesis in WT cancer GC-formed tumor, and smaller tumor border and necrosis tissues were observed in miR-10a KO KGN cells-formed tumor (Fig. 7c). Specifically, PTEN, the previously validated target of miR-10a, was induced in miR-10a KO tumor (Fig. 7d). Two key components of the AKT pathways, phosphorylated AKT (P-AKT) and FOXO1, both were repressed upon miR-10a KO (Fig. 7d). In summary, we showed an oncogenic function of miR-10a in GCT by using another in vivo model.

## Discussion

The issues of tumor recurrence, drug resistance, enhanced invasion, and metastasis remain a challenge in the treatment and the clinical management of various types of tumors. Despite a better understanding of GCT genetic alterations in recent years^15^, the potential of translating these findings into more effective therapy has been limited by the difficulty in identifying functionally relevant drivers of the disease. Because of the inherent heterogeneity and complex nature of GCT, commonly known and highly mutated genes, such as FOXL2^16^, have not been successfully exploited in clinical setting. Hence, identifying molecular drivers of GCT and clarifying the underlying mechanism will be crucial and yet challenging for the development of an alternative therapeutic target especially in advanced-stage cancer.

Here, we identified a miRNA, miR-10a, which is able to regulate PTEN and the Wnt/AKT signaling pathways to promote GCT tumorigenesis and the further downstream events, such as tumor cell survival/apoptosis, proliferation, sensitivity to chemotherapy, migration, and invasion in vitro and in vivo. Several studies have demonstrated the relevance of Wnt/AKT signaling pathways to GCT development. Boerboom et al. found that misregulated WNT/beta-catenin signaling leads to ovarian GCT development^9^. The same group subsequently demonstrated the synergistic effects of PTEN loss and Wnt activation on ovarian GCT development and progression^10^, thereby resulting in increased tumor proliferation and invasion, and a poor patient outcome.

Our present study suggests an upstream event involving miRNA misregulation. Demonstrated through various primary tumor tissues, cancer cell model, and in vivo tumorigenesis, we provided evidences that enhanced expression of miR-10a could confer proliferative and malignant/aggressive traits through modulation of the Wnt and AKT signaling pathways. Furthermore, KO of miR-10a by CRISPR/Cas9 system led to a demise of GCT and downregulation of the two pathways. Importantly, we showed PTEN to be a direct functional target of miR-10a, deeming PTEN as a critical mediator for the oncogenic function of miR-10a.

Several other studies suggest that some miRNAs are critical for the development of chemoresistance. Some miRNAs are differentially expressed in chemosensitive or chemoresistant cells^17^. In this study, we found that forced expression or KO of miR-10a could change the drug (e.g. cisplatin) sensitivity of GCT cells. Moreover, cisplatin significantly reduced endogenous expression of miR-10a. These findings suggest that miR-10a is a determinant of the responsiveness of cancer GC to chemotherapeutic agents. Future studies using a larger cohort might reveal whether or not PTEN/AKT/Wnt and miR-10a could serve as prognostic markers to predict responsiveness to chemotherapy.

Our previous studies showed that miR-10a could induce follicle atresia via affecting development of normal GC^7^. Therefore, miR-10a could be considered as ‘bad guy’ during normal GC growth and development. This negaive effect of miR-10a on normal GC remindered us that miR-10a might play a role in the pathological condition of granulsoa cells, such as GC tumor. In the present study, miR-10a was indeed proven to be an onco-microRNA of GC tumor. The mechanism underlying the different role of miR-10a in physiological and pathological conditions of GC might be the result of different target or related pathway of miR-10a. miR-10a directly targets BDNF and crosstalk with FSH pathway in normal GC; moreover, PTEN is a direct target of miR-10a and AKT/Wnt, whaich are two associated oncogenic pathways in cancerous GC (Supplementary fig. 3). In the future, it is important to decipher the different role of miR-10a in different condtions of GC, which will lay the foundation to understanding the mechanism of GC normal development and carcinogenesis.

## Materials and methods

### FISH analysis of primary human GCT samples

A blinded cohort of 60 primary ovarian granulosa-theca cell tumors (GCT, 50 cases of tumor samples, of which 6 are malignant and 44 are benign; 10 cases of ovarian cyst were also included as control) was purchased from US Biomax. miRCURY LNA miRNA detection probes for miR-10a, was purchased from Exiqon (613307-310, Denmark). FISH was performed according to standard protocol of microRNA FISH from Exiqon.

### Cell culture

Human adult GCT line KGN and human juvenile GCT line Cov434 were obtained from Prof. Zijiang Chen, Shandong University. KGN cells were cultured in DMEM/F12 media (Gibco BRL/Invitrogen, Carlsbad, CA, USA) supplemented with 10% fetal bovine serum and 1% penicillin–streptomycin. Cov434 cells cultured in DMEM media (Gibco, USA) supplemented with 10% fetal bovine serum, 1% NEAA, and 1% penicillin–streptomycin. HEK293T cells were cultured in DMEM media (glutamine contained) supplemented with 10% fetal bovine serum and 1% penicillin–streptomycin. All cells were cultured in a humidified incubator (5% CO_2_) at 37 °C. Cultured cells were dislodged from culture flask using Trypsin (0.05%)/EDTA solution when passaged.

### miRNA mimics and plasmids

All cells were transfected with 20 nM of miR-10a mimics (GenePharma), using Lipofectamine RNAiMAX transfection reagent and Opti-MEMmedium (Life Technologies), according to the manufacturer’s instructions. Control cells were transfected with 20 nM scrambled miRNA (GenePharma). Cells were incubated for 48 h before being subjected to subsequent analysis, unless otherwise mentioned.

MiR-10a precursor sequence and flank sequences were cloned into MDH1-PGK-GFP plasmids (addgene no. 11375) and retrovirus were packaged in HEK293T cells. PTEN shRNAs were designed using the on-line design program from MIT (http://sirna.wi.mit.edu/home.php). The 19-nucleotide hairpin-type shRNAs with a 9-nucleotide loop were cloned into pLVTHM (Addgene, Cambridge, USA). The lentivirus was packaged in HEK293T cell. Green fluorescent protein (GFP) sorting was used to isolate successfully transfected cells. qRT-PCR and Western blot was used for validation of knockdown effect.

### Total and small RNA extraction, reverse transcription, and qPCR

Total RNA was extracted with Trizol reagent (Invitrogen, USA) according to standard protocol. The concentration and quality of all RNA samples were evaluated using Nanodrop 2000 (Thermo, USA), and the 260/280 and 260/230 values of all samples were above 1.8 and 1.9, respectively. Reverse transcription of mRNA was performed with MasterMix kit (Takara, Japan), whereas miRNA reverse transcription was performed with TaqMan reverse transcription kit (Life technology, USA) following the standard manuals. Quantitative PCR of gene was performed using Universal SYBR Green Master mix (Applied Biosystems, USA) and microRNA qPCR was performed using TaqMan-specific microRNA probe (Life technology) on a StepOnePlus real-time PCR system (Applied Biosystems). Gene expression was normalized with GAPDH and miRNA expression was normalized with U6 snoRNA unless otherwise stated.

### Proliferation, apoptosis, invasion, and migration analyses

5 × 10^4^ KGN or Cov434 cells were seeded in 10 cm culture dish for up to 6 days. Proliferation rate was measured using cell-counting kit-8 (DOJINDO) according to the manufacturer’s protocol. Annexin V/propidium iodide (PI) staining was performed for the detection of apoptotic cells. After desired treatment, 1 × 10^6^ cells were collected and washed twice with ice-cold PBS. The cells were then stained using the Alexa Fluor^®^488 annexin V/Dead Cell Apoptosis Kit with Alexa^®^ Fluor 488 annexin V and PI for Flow Cytometry (Invitrogen, CA, USA) according to the manufacturer’s guidelines. The untreated cells served as a negative control for the double staining. Transwell (Costar) was used for migration and invasion assays. Mytomycin C was used for cell synchronization for 24 h. 2 × 10^4^ KGN cells were seeded into the upper chamber with serum-free medium and serum-contained medium was added to the lower wells (Matrigel was coated in upper chamber for invasion assay). After 24 and 48 h, the upper chamber was washed with PBS twice and fixed by 4% paraformaldehyde for 20 min, and then 1% crystal violet was used to stain migrated and invasive cells.

### ELISA

1 × 10^5^ cells were seeded in 12-well plate for 48 h. Supernatant was collected for secreted estrone and estrodial measurement. Estrone was detected by human Estrone competitive ELISA kit (Invitrogen) and estradiol was detected by Estradiol human ELISA kit (Thermo).

### Cisplatin and taxol treatment

Cultured KGN cells were treated with different concentrations of cisplatin (2.5–7.5 μM) or taxol (5–10 mM) for 48 h. At the end of the experiments, the number of live cells was determined by CCK-8 assays. Cisplatin-treated KGN cells were also collected for endogenous miR-10a detection by RT-qPCR assay.

### Immunofluorescence

30% sucrose-dehydrated and OCT-embedded tumor tissue samples were sliced using Cyrost (Leica CM3050 S). The slices were washed once with PBS and fixed with 4% paraformaldehyde/4% sucrose in PBS at room temperature, followed by permeabilization and DNA denaturation by 0.2% TritonX-100 in 4 M HCl. After that, the cells were washed with PBS and blocked with 80 μL BSA (3%). The slides were incubated with primary antibody of anti-GSK-3β (Abcam), anti-β-Catenin (Abcam) in BSA (3%) at 4 °C overnight, and then conjugated with and Hoechst 33342 or DAPI.

### IHC assay

Paraffin-embedded KGN cells-formed tumor were sliced and then blocked in 5% goat serum for 30 min. The tissue was incubated in primary anti-PTEN (Abcam), anti-P-Akt (Cell signaling), and anti-FOXO1 (Abcam) antibodies overnight at 4 °C. The IHC staining was generally performed as reported previously^18^.

### RNA-seq and data analysis

Total RNA was quantified using a NanoDrop2000 spectrophotometer (NanoDrop Technologies, Wilmington, DE). KGN cells were collected 48 h after transfection of miR-10a mimics and negative control. For detection of whole transcriptome expression, RNA-sequencing was performed by GROKEN Bioscience (China) according to standard procedure.

### Western blot

Cells were lysed in SDS buffer. The protein concentration was measured by BCA assay kit (Thermo). Equal amounts of cell lysates were loaded, blotted onto a PVDF membrane, and probed with the following primary antibodies: anti-PTEN(Cell signaling), anti-Akt(abcam), anti-p-Akt(Cell signaling), anti-PI3K(Abcam), anti-TSC1(Cell signaling), anti-TSC2(Cell signaling), anti-S6K(Cell signaling), ant-mTOR(Abcam), anti-FOXO1(Abcam), anti-GSK3β(Cell signaling), anti-p-GSK3β(Cell signaling), anti-β-Catenin(Cell signaling), anti-p-β-Catenin(Santa Cruz), anti-GAPDH (Cell signaling), and anti-β-Actin (Santa Cruz) was used as loading controls. After incubation with the appropriate secondary antibodies, signals were visualized by enhanced chemiluminescence (GE systems).

### Luciferase reporter assay

HEK293T cells grown in 24-well plates were transfected with 50 nM miR-10a mimics (GenePharma, China), 100 ng of pmirGLO vector (Promega, USA) tagged with 3′UTR that includes miR-10-binding sites or empty pmirGLO plasmid by using Lipofectamine 2000 (Invitrogen, USA). The Firefly and Renilla luciferase activities in the cell lysates were assayed with a Dual-Luciferase Reporter Assay System (Promega) at 48 h post-transfection.

### CRISPR-Cas9 inducible KO of miR-10a

For the inducible sgRNA constructs, the previously described pFH1tUTG plasmid^19^ was modified to contain the H1-Tet-sgRNA cassette, which was inserted into Pac I sites upstream of the hUbiquitin promoter. This sequence contains two bi-directional BsmbI sites for subsequent cloning of different sgRNA sequences. The constitutive sgRNA and Cas9 expression vectors were derived from the pFUGW, which has been described previously^20^. For the constitutive sgRNA expression, the H1t promoter was inserted into the Pac1 site upstream of the hUbiquitin promoter. For the Cas9 expression vector, the eGFP cassette was replaced by the recently described Cas9^21^ linked via the T2A peptide to the mCherry fluorescent reporter protein. The MIT CRISPR design software was used for the design of sgRNAs (http://crispr.mit.edu). To clone individual miR-10a sgRNAs, 24-bp oligonucleotides containing the sgRNA sequences (sequence to be provided) were synthesized (Invitrogen). They included a 4-bp overhang for the forward (TCCC) and complementary reverse (AAAC) oligos to enable cloning into the BsmbI sites of the lentivector.

### Xenograft of cancer GC in nude mice

All in vivo experiments were performed according to approved protocols from the Animal Research Committee at the LASEC, Chinese University of Hong Kong. For the subcutaneous model, dox-inducible miR-10a KO KGN cells were injected s.c. (5 × 10^6^ cells/mice), in 4-week-old to 6-week-old female nude mice. Group A mice were fed with dox-contained water for one week. All mice were killed after 8 weeks and KGN-formed tumor were extracted and weighed to compare relative tumor burden. RNA and protein from tumor tissue was extracted as described above. All tumors were subsequently analyzed by haematoxylin and eosin staining and by immunohistological staining.

### Orthotopic model

pLVTHM KGN cells and pLVTHM-miR10a KGN cells (1 × 10^6^) were injected under the ovarian bursa in 4–6-week-old female nude mice. All mice were killed after 4 weeks. Then ovarian nodules were extracted for consequent analysis.

### Statistics

The error bars represent the standard error of mean (SEM) of three independent experiments. *, **, and *** indicate *P* < 0.05, *P* < 0.01, and *P* < 0.0001, respectively (Student’s *t*-test).

## Electronic supplementary material

Supplementary figure 1

Supplementary figure 2

Supplementary figure 3
