# Supplementary figures and images for: MicroRNA-10a promotes granulosa cells tumor development via PTEN-AKT/Wnt regulatory axis
Source: Cell Death Dis. 2018 Oct 22;9(11):1076. doi: 10.1038/s41419-018-1117-5 (PMC6197200; doi:10.1038/s41419-018-1117-5)

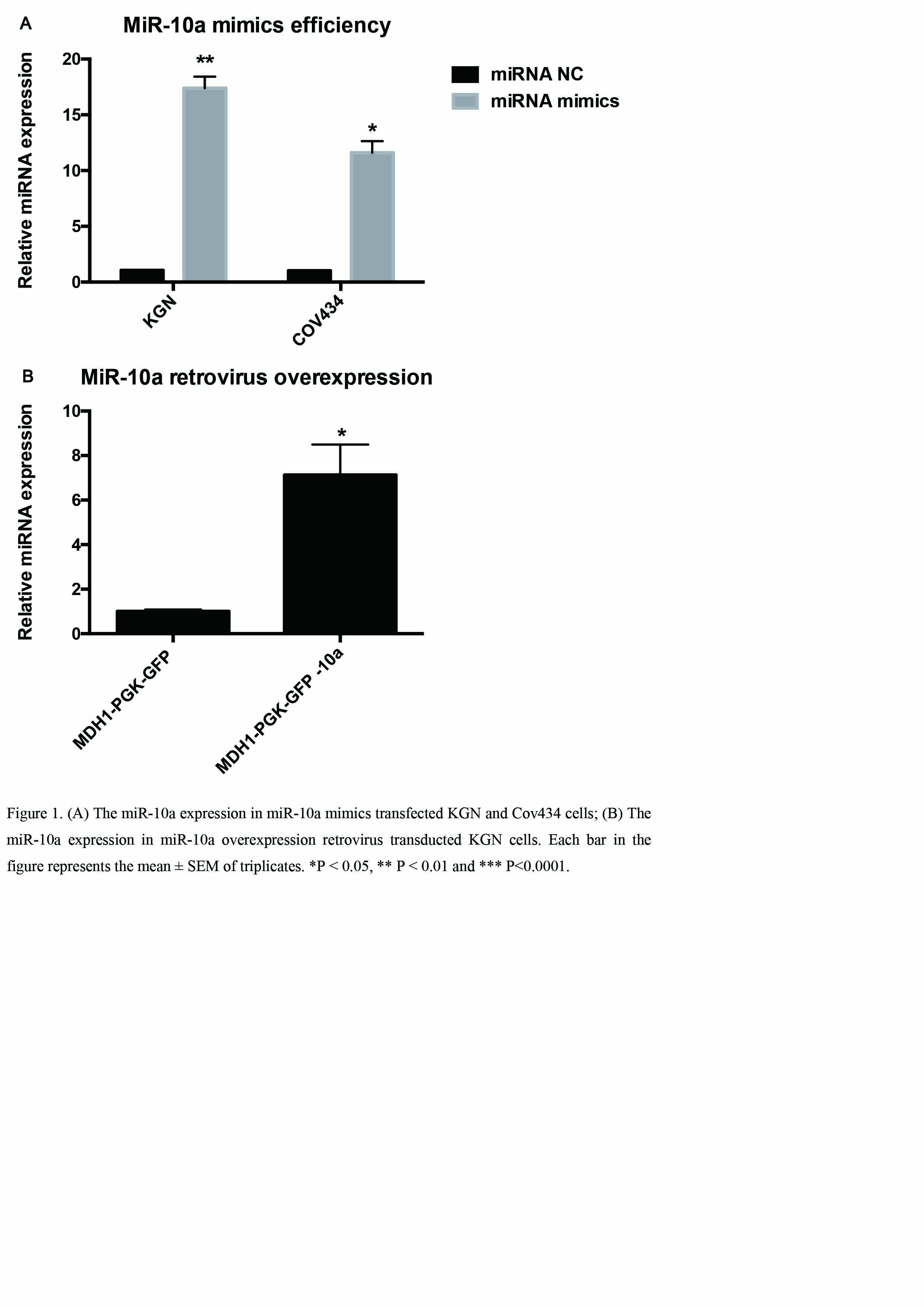

Supplement: Supplementary file 1 — Supplementary figure 1 [file 41419_2018_1117_MOESM1_ESM.jpg]

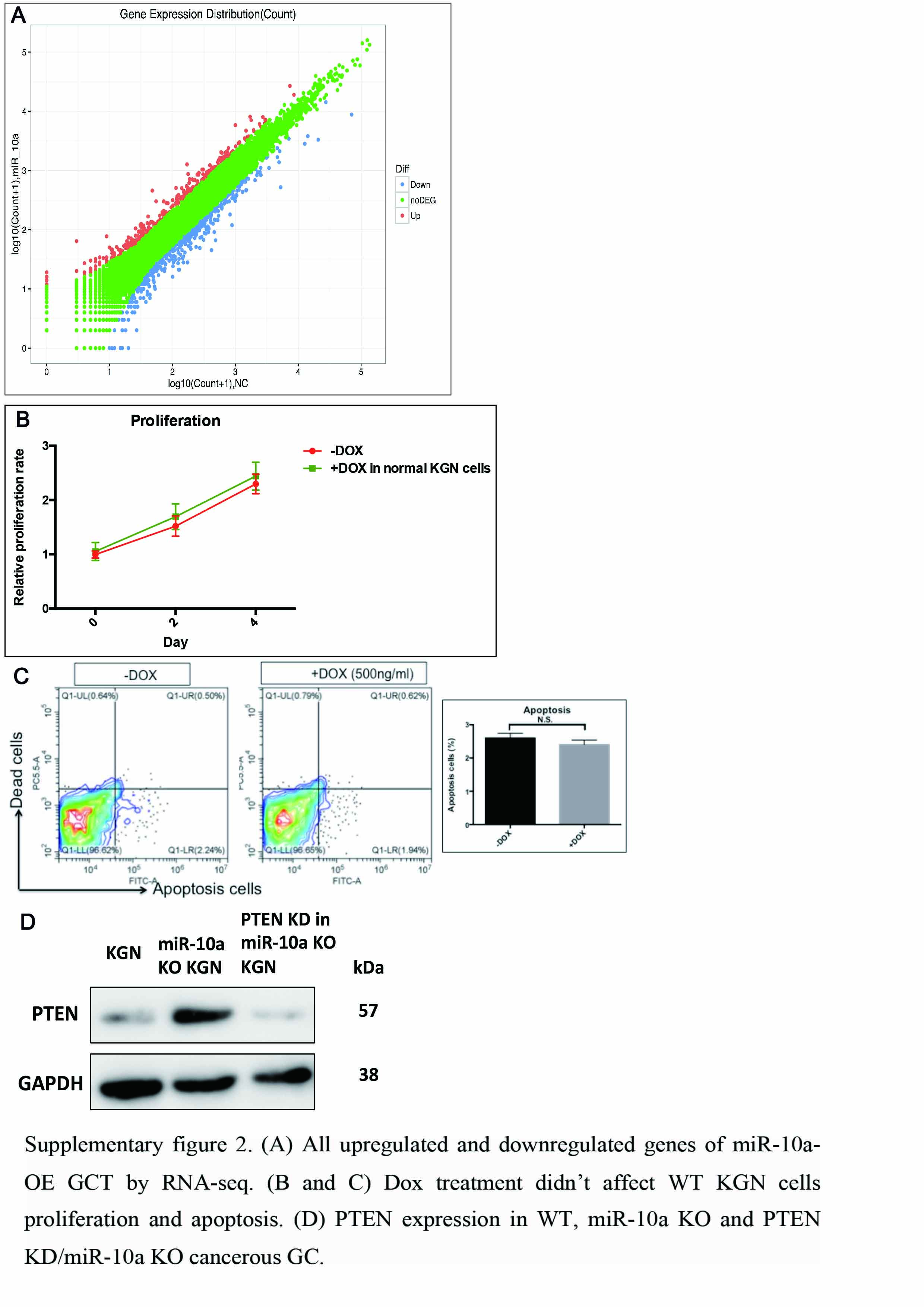

Supplement: Supplementary file 2 — Supplementary figure 2 [file 41419_2018_1117_MOESM2_ESM.jpg]

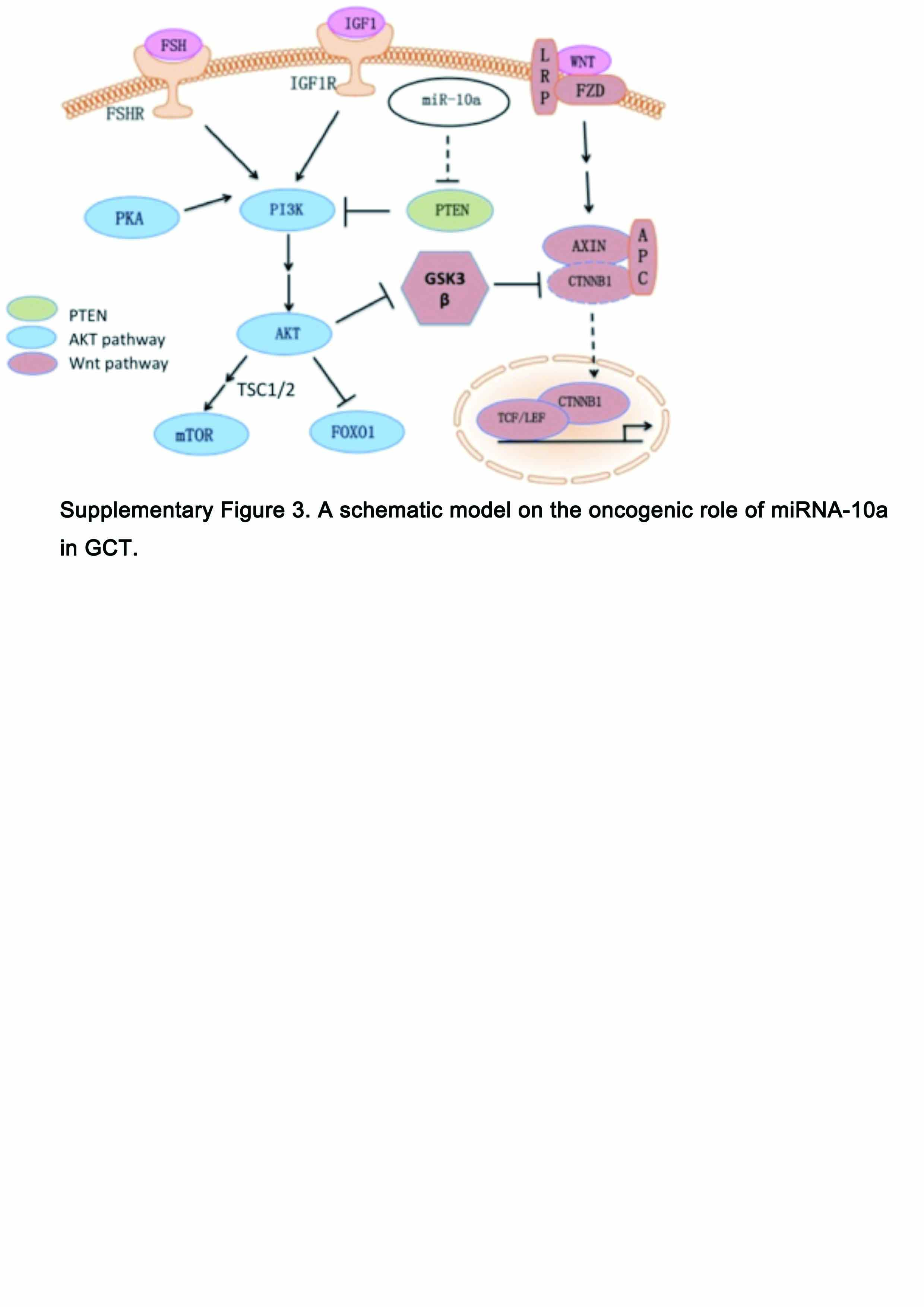

Supplement: Supplementary file 3 — Supplementary figure 3 [file 41419_2018_1117_MOESM3_ESM.jpg]
